# Single cell RNA analysis of the left–right organizer transcriptome reveals potential novel heterotaxy genes

**DOI:** 10.1038/s41598-023-36862-2

**Published:** 2023-07-01

**Authors:** Helen M. Bellchambers, Amruta R. Phatak, Mardi J. Nenni, Maria B. Padua, Hongyu Gao, Yunlong Liu, Stephanie M. Ware

**Affiliations:** 1 Herman B Wells Center for Pediatric Research, Department of Pediatrics, Indiana University School of Medicine, 1044 W. Walnut Street, Indianapolis, IN 46202 USA; 2 Division of Developmental Biology, Cincinnati Children’s Hospital Medical Center, Cincinnati, OH 45229 USA; 3 Department of Medical and Molecular Genetics, Indiana University School of Medicine, Indianapolis, IN 46202 USA

**Keywords:** Congenital heart defects, Embryology

## Abstract

The establishment of left–right patterning in mice occurs at a transient structure called the embryonic node or left–right organizer (LRO). Previous analysis of the LRO has proven challenging due to the small cell number and transient nature of this structure. Here, we seek to overcome these difficulties to define the transcriptome of the LRO. Specifically, we used single cell RNA sequencing of 0–1 somite embryos to identify LRO enriched genes which were compared to bulk RNA sequencing of LRO cells isolated by fluorescent activated cell sorting. Gene ontology analysis indicated an enrichment of genes associated with cilia and laterality terms. Furthermore, comparison to previously identified LRO genes identified 127 novel LRO genes, including *Ttll3*, *Syne1* and *Sparcl1*, for which the expression patterns were validated using whole mount in situ hybridization. This list of novel LRO genes will be a useful resource for further studies on LRO morphogenesis, the establishment of laterality and the genetic causes of heterotaxy.

## Introduction

Establishing the left–right (LR) axis during early embryogenesis is critical for placement and patterning of the heart and visceral organs. In the mouse embryo, the first molecular evidence of LR asymmetry appears at the ventral LRO^1^. The LRO contains two distinct cell types: the central cells, referred to as pit cells, and the outer cells referred to as crown cells. Both cell types contain a single cilium on the ventral surface of the cell, but the pit cilia rotate whereas the crown cilia are immotile.

At the early bud stage, pit cell cilia are located in the center of cell, thus the rotation produces a random extracellular fluid flow. From late head fold (LHF) to the three-somite stage, the cilia move towards the posterior of the cell induced by planar cell polarity signaling cues^2–5^. The posterior and ventral position of cilia combined with the dome shape of cells then creates a leftward flow across the LRO, which has been detected in vivo using particle image velocity analysis at the one somite stage^3^. Crown cells at the edge of the LRO are thought to sense this fluid flow through immotile cilia; however, the exact mechanism by which this occurs is unclear. Two mechanisms have been proposed: the first suggests that the fluid flow creates a gradient of either determinant particles or morphogens^6,7^; the second, which is strongly supported by two excellent recent papers^8,9^, suggests that the immotile cilia on the crown cells sense the flow and react by releasing Ca^2+^ ions from the cilium into the cytoplasm of crown cells^10–12^.

Regardless of the mechanism, the result is that *Cerl2/Dand5* mRNA is degraded in crown cells on the left side of the LRO, thus creating a right side biased expression pattern^13^. The asymmetric expression of *Cerl2/Dand5* has been detected by the LHF stage, and thus is the first gene known to be asymmetrically expressed across the LR axis. *Cerl2/Dand5* is a *Nodal* antagonist, thus this right side dominant expression pattern in turn creates a left side asymmetric expression of *Nodal* in the crown cells, which is then transferred to the left lateral plate mesoderm (LPM) in a manner that is not well understood but involves the juxtaposed endoderm cells^14–16^. These signaling events are highly dynamic and occur within a few hours of development.

The genetic causes of congenital heart defects (CHD) are often unknown and molecular pathways important for human cardiac development remain to be identified. Mutations in genes critical for LR patterning have been identified in patients with cardiac laterality disorders as well as isolated CHD^17–19^. The heart begins as a linear tube and then undergoes asymmetric looping using cues initiating along the LR axis. Consequently, malformations of the heart often occur when the LR axis is not properly established^20,21^. Other organs are also affected by the LR axis; thus these heart defects can co-occur with other organ laterality defects which are collectively classified as heterotaxy^22^. Patients with heterotaxy, a disorder of the LR axis, account for at least 3% of all CHD^23^ but the genes and developmental mechanisms may contribute to a larger number of CHD cases since cardiac phenotypes seen in heterotaxy such as transposition of the great arteries or double outlet right ventricle more commonly occur without evidence of other visceral situs abnormalities.

Because of the essential role of cilia in establishing initial LR asymmetry, many known critical cilia genes are associated with heterotaxy. For example, motile cilia required for extracellular fluid flow are under the regulation of the transcription factor, *Foxj1*^24,25^, thus in the absence of *Foxj1*, cilia fail to form and function properly, resulting in laterality defects in animal models^24–29^.

Despite the importance of proper LRO and cilia formation and function, there are still many unanswered questions due to the technical challenges of studying LRO development and signal propagation. These include, but are not limited to, the transient nature of the LRO, a limited cell number, and difficulties in early embryonic manipulations. Previously, a large-scale approach to identify markers of organizer tissue was performed using microarray in whole mouse embryos^30^. The authors compared normal embryos with mutants lacking gastrula-organizing structures, including the LRO, to identify genes dysregulated during abnormal gastrulation. Another paper spatially resolved embryos by dividing gastrulation stage embryos into geographically defined regions^31^. As the LRO is known to be present at the distal tip of the embryo at E7.5, the authors where able to determine which transcriptome included the LRO/node cells based on its spatial–temporal location. However, the study focused on the gastrulation stage embryos and thus does not define the LRO transcriptome at the point of LR determination.

Recent advances in technology have enabled transcriptomic analysis at the single cell level. In particular, Pijuan-Sala et al.^32^ generated single cell maps of mouse gastrulation and early organogenesis. However, given the small number of cells in an embryo and the limitations of the technology, this study required pooling of multiple embryos to obtain sufficient cells for analysis. Since that time, the technology has further improved to enable fewer input cells and therefore a single embryo can be analyzed individually. Here, we used single cell RNA sequencing (scRNA-seq) to generate a transcriptomic profile list of LRO genes from precisely staged 0–1 somite mouse embryos, when the LRO fluid flow is first detected and both *Dand5* and *Nodal* are becoming asymmetrically expressed in the crown cells. These genes were compared to a dataset of genes derived from bulk RNA sequencing (RNA-seq) of fluorescently labeled LRO cells isolated by fluorescent activated cell sorting (FACS) from *FOXJ1*-*EGFP* transgenic mouse embryos. As expected, this LRO gene list contained many genes previously associated with heterotaxy. In addition, novel LRO genes were identified, providing a resource for the research community, especially those studying LR patterning, cilia and heart development, and paving the way for future functional studies.

## Results and discussion

### scRNA-seq analysis of LRO cell genes

**Figure 1:**
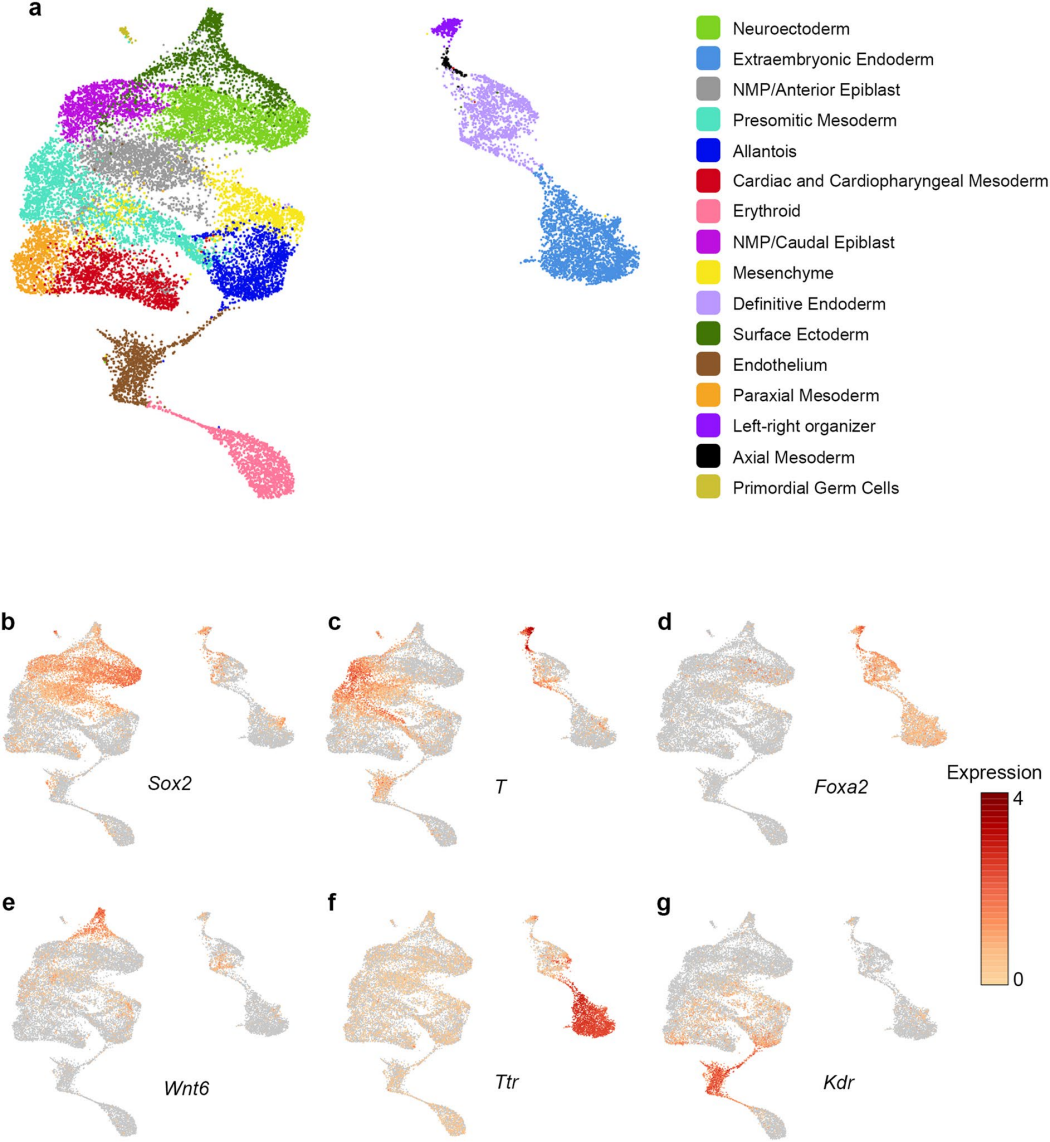
Distinguishing cell cluster identities of 0–1 somite embryos. (**a**) Unifold manifold approximation and projection (UMAP) plot of 21,552 cells from n = 3 embryos. 16 clusters were identified based on the expression of highly variable genes. (**b**–**g**) Feature plots displaying expression of known marker genes, including (**b**) the ectoderm marker *Sox2*, (**c**) the mesoderm marker *T*, (**d**) the endoderm marker *Foxa2*, (**e**) the surface ectoderm marker *Wnt6*, (**f**) the extraembryonic endoderm marker *Ttr* and (**g**) the endothelium marker *Kdr*. Expression, log normalized expression; NMP, Neuromesodermal progenitors.

To characterize the LRO transcriptome, we generated single-cell gene expression profiles of dissociated cells from three individual wildtype embryos specifically selected based on the morphology of the embryo to be at the 0–1 somite stage. A total of 23,533 single cells were obtained, of which 21,552 passed quality control measures and a median of 5079, 3663 and 3949 genes were detected per cell for the first, second and third embryo, respectively. As all embryos were male (as determined by genotyping), we examined expression of the female specific transcript *Xist* and the Y-chromosome genes *Ddx3y*, *Eif2s3y* and *Uty*. Cells showed expression of the male specific genes, with no expression of the female specific *Xist* (Supplementary Fig. S1), indicating there was no maternal contamination. By unsupervised clustering, we were able to distinguish 16 clusters (Fig. 1a). To determine the identity of these clusters, we first examined expression of general markers of ectoderm (*Sox2*, *Dlx5*), mesoderm (*T*, *Aldh1a2*) and endoderm (*Spink1*, *Sox17* and *Foxa2*) to roughly divide the cells into these three cell types (Fig. 1b–d). Within each of these subtypes, clusters were assigned based on known marker genes, such as *Wnt6* for the surface ectoderm, *Ttr* for the extraembryonic endoderm and *Kdr* for the endothelium (Fig. 1e–g, Supplementary Figs. S2–S3, Supplementary Tables S1–S3, and Supplementary Methods).

**Figure 2:**
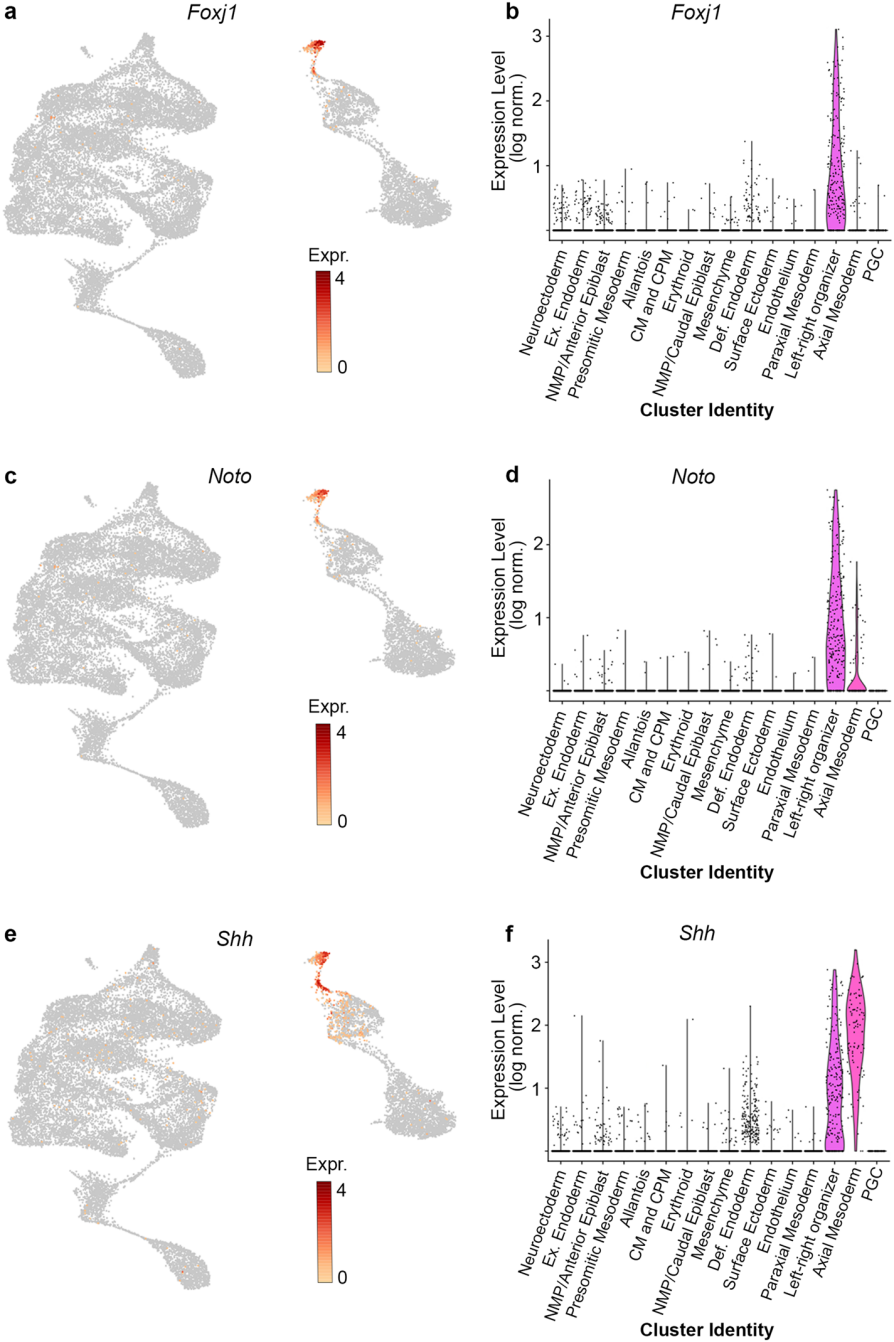
Identification of left–right organizer (LRO)/notochord clusters. Feature plots of *Foxj1* (**a**), *Noto* (**c**), and *Shh* (**e**). Each dot represents a single cell. Violin plots of *Foxj1* (**b**), *Noto* (**d**), and *Shh* (**f**) expression in the LRO (light purple) and axial mesoderm (dark pink) clusters. Each dot represents the log normalized expression value (y-axis) of a single cell of a particular cluster (x-axis). Expr, log normalized expression; Ex, extraembryonic; NMP, Neuromesodermal progenitors; CM, Cardiac mesoderm; CPM, Cardiopharyngeal mesoderm; Def, Definitive; PGC, Primordial germ cells.

**Figure 3:**
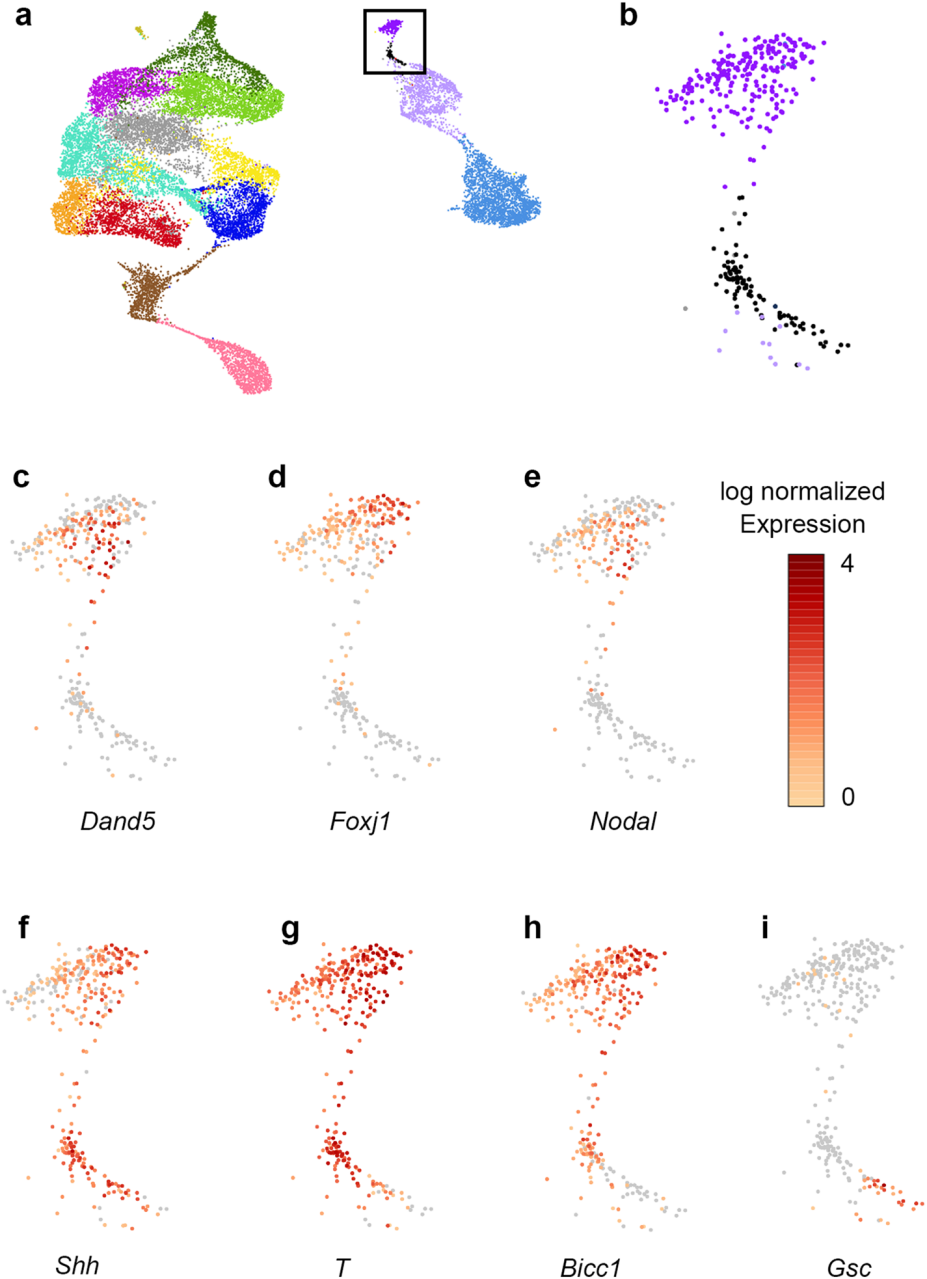
Gene expression within the left–right organizer (LRO)/axial mesoderm clusters. (**a**) UMAP with LRO/axial mesoderm clusters marked by a black box. (**b**) Magnified view of the black squared box from (**a**). (**c**–**i**) Expression of different genes within the LRO cluster, including the LRO specific genes (**c**) *Dand5*, (**d**) *Foxj1* and (**e**) *Nodal*, the LRO/notochord genes (**f**) *Shh*, (**g**) *T* and (**h**) *Bicc1*, as well as the prechordal plate gene (**i**) *Gsc*. Purple = cluster 13/LRO cluster; Black = cluster 14/axial mesoderm cluster.

Two clusters (clusters 13 and 14; purple and black clusters, respectively, in Fig. 1a) showed expression of both the endoderm marker *Foxa2* and the mesoderm marker *T*, suggesting these clusters represent the mesendoderm cells of the LRO and notochord (Fig. 1c–d). Examination of the LRO/notochord genes *Foxj1*, *Noto* and *Shh* confirmed this identity (Fig. 2). To understand the distinction between these clusters, they were examined for markers known to be restricted to the LRO or to be expressed in both LRO and notochord (Fig. 3). Only cluster 13 showed expression of previously identified LRO specific genes including *Dand5*, *Foxj1* and *Nodal* (Fig. 3c–e), thus it was designated as the LRO cell cluster. Cluster 14 lacked these genes, but both clusters showed expression of *Shh*, *T* and *Bicc1* (Fig. 3f–h), which are expressed in both LRO and notochord populations. The presence of these notochord/LRO genes combined with the lack of LRO genes suggests these are notochord cells. However, a subset of cells in cluster 14 also had expression of *Gsc* (Fig. 3i), a known marker of prechordal plate, which lies at the midline of the embryo directly anterior of notochord. As cluster 14 contained both notochord and prechordal plate cells it was collectively defined as the axial mesoderm.

**Figure 4:**
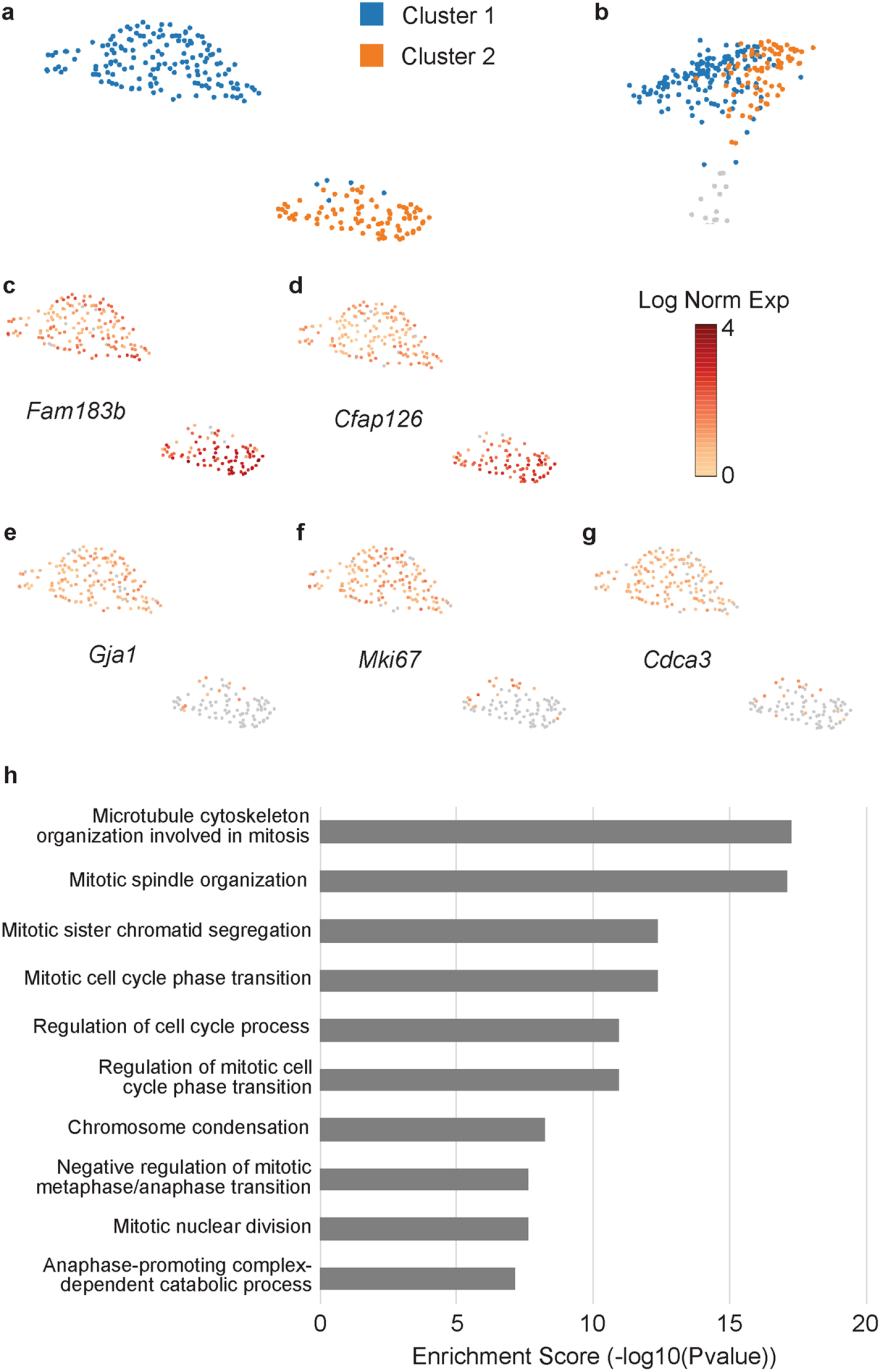
Identification of left–right organizer (LRO) cell subtypes. (**a**) Subclusters within the LRO cluster. (**b**) Position of the subclusters within the original LRO cluster. (**c**–**g**) Expression of different genes within the LRO subclusters, including the LRO genes (**c**) *Fam183b*, (**d**) *Cfap126*, (**e**) as well as *Gja1*, (**f**) *Mki67*, and (**g**) *Cdca3* mitosis and cell cycle-related genes. (**h**) Gene ontology analysis of the cluster 1 specific genes.

To further delineate these cells, we performed sub-clustering of the LRO cluster, which resolved into two subclusters (Fig. 4a,b). Seurat was used to identify differentially expressed genes. Several genes showed higher expression in sub-cluster 2, with known LRO genes *Fam183b* and *Cfap126* displaying the highest fold change (Fig. 4c,d and Supplementary Table S4). However, none of the differentially expressed genes were specific to this cluster. On the other hand, sub-cluster 1 showed several genes distinct from sub-cluster 2, including *Gja1*, *Mki67* and *Cdca3* (Fig. 4e–g and Supplementary Table S5). Gene ontology (GO) enrichment was performed on the genes most specific to cluster 1 (defined by pct.1-pct.2 > 0.6 cut-off, see methods). The most enriched terms were for mitosis and/or cell cycle (Fig. 4h and Supplementary Table S6), suggesting the difference between these clusters may be due to cell cycle and not distinct cell types within the LRO.

### Comparison of bulk RNA-seq and scRNA-seq LRO specific genes

The LRO transcriptome was also analyzed by bulk RNA-seq for scRNA-seq gene validation. To isolate these cells for RNA-seq, the EGFP expressing LRO cells as well as the surrounding EGFP negative non-LRO cells from *FOXJ1-EGFP* mouse embryos were sorted using FACS (Supplementary Fig. S4). LRO and non-LRO transcripts were quantified in reads per kilobase per million (RPKM) to normalize for length of RNA and sequencing depth between samples using Partek^®^ Genomics Suite^®^ software which also determined fold-change and q-values. To define genes restricted to the LRO we limited genes to those with 0 RPKM in the non-LRO cells and a q-value < 0.01. We further limited the genes to those with a > 1 RPKM in the LRO cells, which gave a final list of 17 LRO specific genes (Supplementary Table S7). We used the scRNA-seq data to generate expression and violin plots of these 17 genes to determine their expression patterns (Supplementary Fig. S5). Twelve of these genes were specific to the LRO whereas two were largely restricted to the LRO but also had some expression in the axial mesoderm (Supplementary Table S7). No expression was detected for the remaining three in any cluster, which is likely due to differences in sensitivity between the two assays (Supplementary Table S7). These results collectively show there is consistency between the genes detected via bulk RNA-seq and scRNA-seq, indicating that scRNA-seq can be used to identity LRO specific genes.

### Generation of the LRO gene list

A list of differentially expressed LRO genes was generated with Seurat (Supplementary Table S8). In order to restrict our list to highly enriched LRO genes, we filtered the initial gene list using more stringent cut-offs to remove genes with expression in other clusters. The cutoffs were chosen based on 27 previously published LRO specific genes (Supplementary Table S9). After filtering the Seurat gene list based on these cut-offs, 196 genes remained and were defined as the LRO transcriptome (Supplementary Table S10).

**Figure 5:**
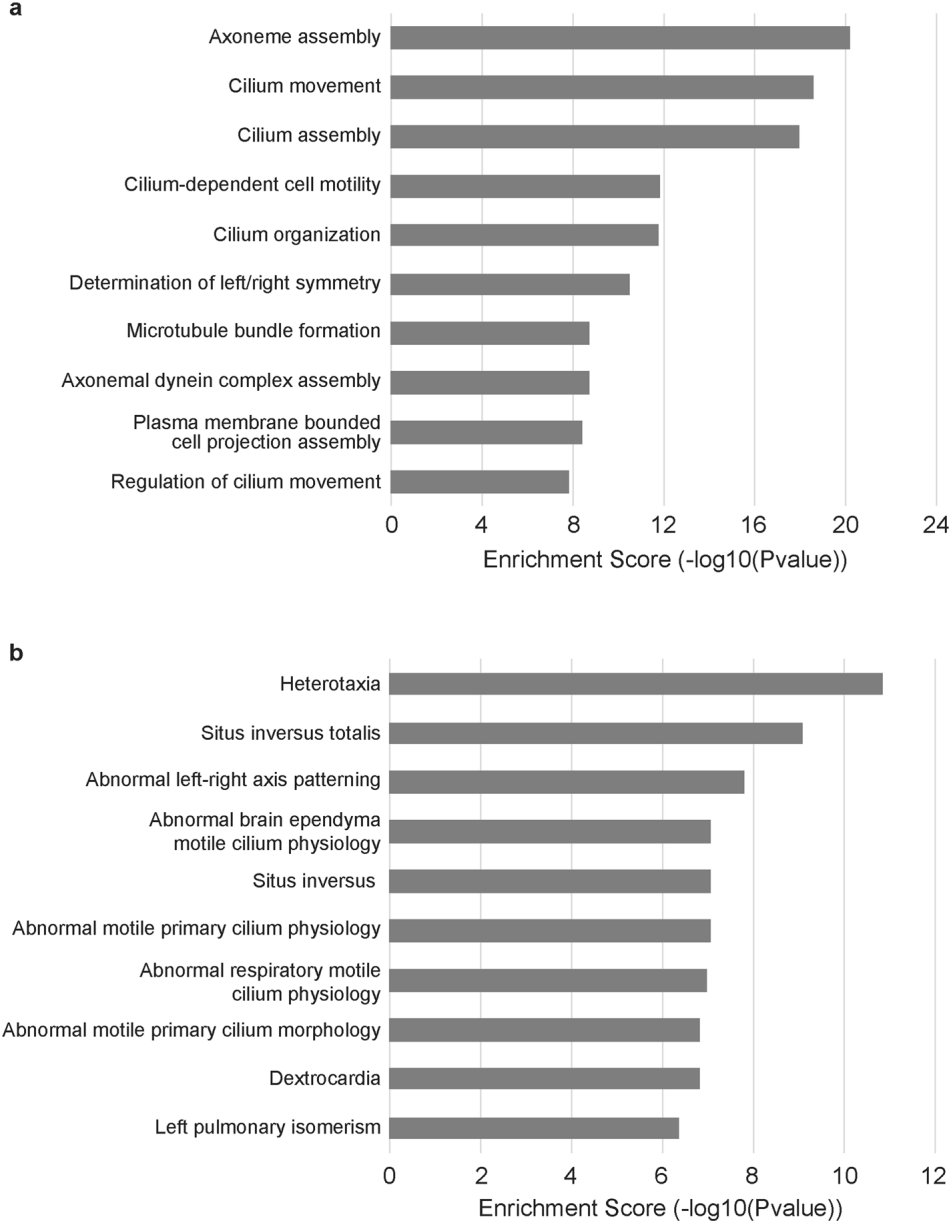
Gene Ontology analysis of left–right organizer (LRO) gene list. (**a**) Top ten enriched biological process terms. (**b**) Top ten enriched mammalian phenotype terms.

We performed GO enrichment to determine which biological processes were statistically enriched in the LRO gene list. Consistent with the known importance of cilia for LRO function, 9 out of the 10 of the biological process terms with the highest enrichment score were related to cilia assembly or movement (Fig. 5a and Supplementary Table S11). To understand the functional relevance of the gene list, we also performed GO enrichment for the mammalian phenotypes that were statistically enriched in the LRO gene list (Fig. 5b and Supplementary Table S12). Of the 10 most enriched terms, the majority were previously linked to abnormal establishment of LR asymmetry, such as *situs inversus* and heterotaxia, or terms linked to phenotypes found in patients with heterotaxy syndrome, such as dextrocardia and left pulmonary isomerism. The remaining terms were phenotypes linked to abnormal cilia phenotypes, likely due to the critical role of cilia for LRO function. The large number of terms associated with heterotaxy together with the known role of the LRO in establishment of LR patterning suggested that the LRO gene list could be a source of novel heterotaxy genes. We therefore compared this list to a recently compiled list of known laterality defect genes^19^ as well as genes listed in the mouse genome database as having the annotated phenotype of ‘heterotaxia’ or ‘abnormal left–right patterning’ in the mammalian phenotype browser^33^. We found 28 genes in the list have previously been associated with heterotaxy or *situs inversus* in humans and 31 have been associated with either heterotaxia or abnormal LR patterning in mice. Due to a 19 gene overlap between the human and mouse phenotypes this gave a total 40 genes previously associated with heterotaxy in humans or mice (Supplementary Table S13). The remaining 156 genes have not been previously associated with heterotaxy in humans or mice to our knowledge.

**Figure 6:**
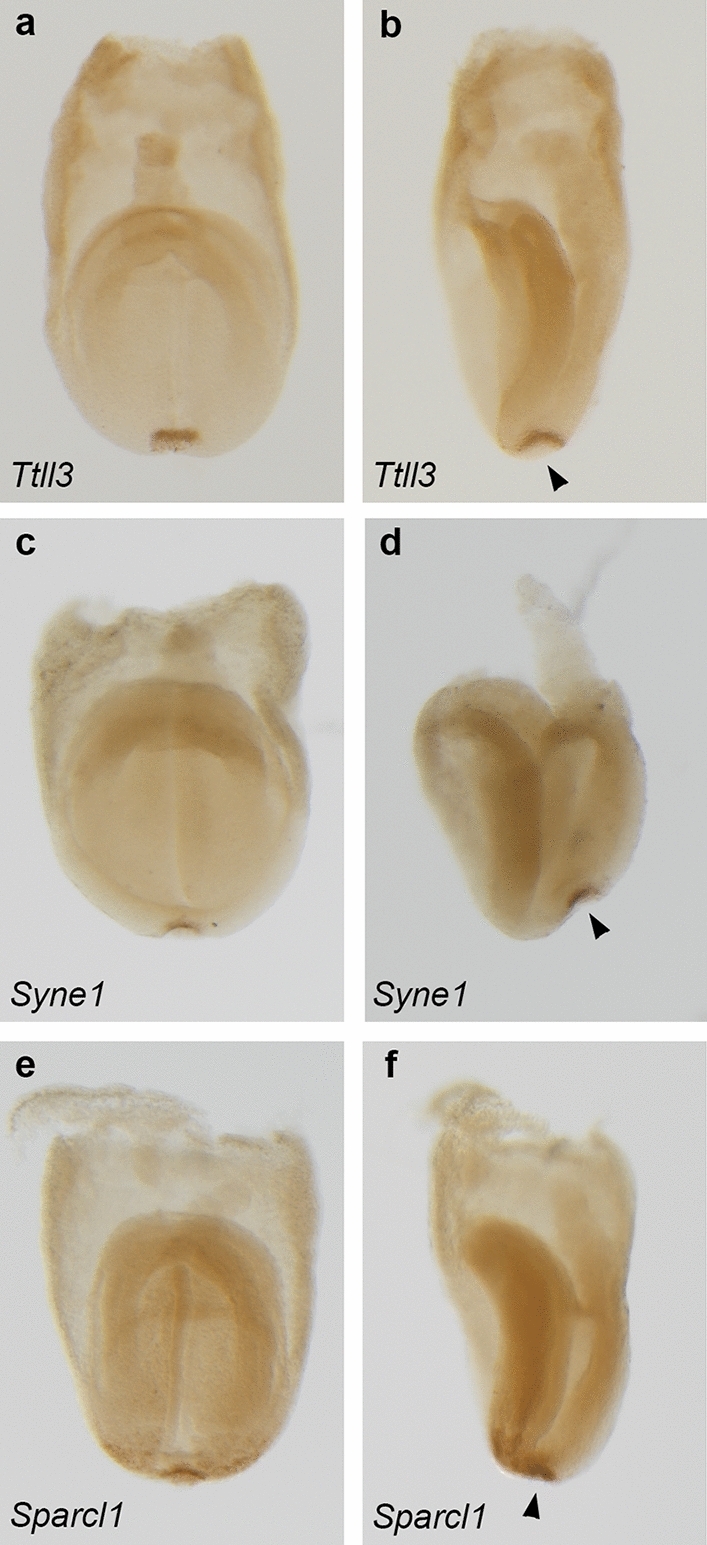
RNAscope in situ hybridization of novel left–right organizer (LRO) genes. (**a**–**d**) Whole mount in situ hybridization of 0–1 somite embryos showing expression (dark brown) of (**a**–**b**) *Ttll3* **(**n = 3), (**c**–**d**) *Syne1 * (n = 5) and *Sparcl1 * (n = 5) in the LRO. (**a**, **c**, **e**) Frontal view and (**b**, **d**, **f**) lateral view of embryos. Arrowheads point to LRO.

To understand which of these genes were truly novel LRO genes, we compared our list of 196 LRO genes to three previous sources of LRO genes (Supplementary Table S13). First, we manually examined expression of all 196 genes at E7.5–8.5 in the mouse Gene Expression Database (GXD)^34^ and thus identified 44 genes with previous published in situ staining in the LRO. Second, we compared our 196 genes to a list of LRO/notochord genes that has previously been generated via microarray comparison of wildtype and *Foxa2* mutant embryos^30^. Of the 20 LRO/notochord genes identified in the paper, 12 were found in our LRO gene list. Examination of the remaining eight genes in GXD showed that the other genes were not specific to the LRO, with additional regions of expression in the midline or extraembryonic tissues. Third, we compared our list to a paper that examines the spatial transcriptome of gastrulation stage embryos and defines LRO specific genes based on the assumption that the LRO is located at the distal tip of the embryo at E7.5^31^. Of the 50 LRO specific genes identified in that paper, 39 were present in our gene list. Of the remaining eleven, only *Smoc1* had data in the GXD at the correct stage and it was expressed in the LPM in addition to the LRO. As there is overlap between these three methods of identifying gene lists, overall, there were 69 genes that have previously been defined as LRO genes. We have therefore identified 127 potential novel LRO genes (of which 19 have previously been associated with heterotaxy in humans/mice and the remaining 108 have not been associated with heterotaxy or other laterality defects). Whole mount in situ hybridization confirmed the expression of some of these novel genes in the LRO including *Ttll3*, *Syne1,* and *Sparcl1* (Fig. 6).

**Figure 7:**
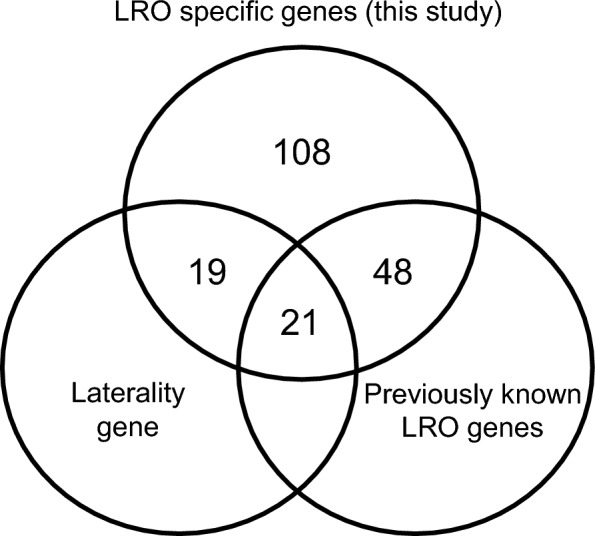
Venn diagram of left–right organizer (LRO) gene list. The genes previously associated with laterality defects (either heterotaxy or *situs inversus*) or identified as LRO specific genes.

It is worth noting that not all known heterotaxy/laterality genes were previously identified LRO genes (Fig. 7). For example, *Ccdc114*, *Dnaaf3* and *Lrrc56*, have previously been associated with heterotaxy or *situs inversus*^35–38^, but the expression patterns for these genes at the onset of LR asymmetry has not been examined. Thus, the finding of LRO expression might be useful for uncovering the mechanistic basis by which these genes affect LR patterning.

The LRO has been challenging to study due to this structure’s transient nature, small size, and low cell number. As such, despite the critical role of the LRO for establishing LR asymmetry and the known association of genes critical for structure/function of the LRO with heterotaxy, the LRO transcriptome has been poorly characterized. Improvements in technology have enabled us to improve our understanding of this structure by using scRNA-seq to examine the LRO transcriptome and thus identify 127 novel LRO genes. Given the enrichment of known heterotaxy genes within our LRO gene list, as well as the link between establishment of LR asymmetry and heterotaxy, this list represents a potential new source of candidate heterotaxy genes. Indeed, while this paper was under review, mutation of *TTC12* (one of our LRO expression genes) was identified in humans with laterality defects and validated using a zebrafish model^39^. Thus, this gene list will be a useful resource for the research community, particularly for those studying LRO morphogenesis and establishment of LR asymmetry.

## Methods

### Mice, embryo collection and genotyping

For the scRNA-seq and RNAscope experiments, mice were housed in the AAALAC accredited Indiana University School of Medicine Animal Facility. Embryos were harvested at E8.0 in ice-cold phosphate buffered saline (PBS). For the scRNA experiments, PBS was supplemented with 10% (v/v) of fetal bovine serum (FBS/PBS). Embryos aged between 0 and 1 somite were selected for further analysis and the extraembryonic tissue was removed for DNA extraction and genotyping. Briefly, the extraembryonic tissue from each embryo was divided into two pieces. Half of this tissue was lysed using the Hotshot lysis buffer^40^ for 10 min at 95 °C. The other half was lysed using the Cells-to-Ct 1-Step TaqMan kit (ThermoFisher, Carlsbad, CA, USA) following the manufacturer’s instructions. Both DNA samples were genotyped using the *Zic3* genotyping assay previously described^41^, which includes *Sry* primers for sex determination.

For FACS experiments, mice were housed in the AAALAC accredited Cincinnati Children’s Hospital Research Foundation Animal Facility. The *FOXJ1*-*EGFP* mouse line^42^ was a gift from Dr. Kenny Campbell, Cincinnati Children’s Hospital Medical Center (CCHMC). Embryos were harvested at E8.25 in ice-cold FBS/PBS.

Mice were maintained on a C57BL/6 × 129 SvEv background and all experiments were approved by the Institutional Animal Care and Use Committee (IACUC). All experiments were performed in accordance with IACUC guidelines and regulations. Authors complied with ARRIVE guidelines.

### Single-cell sample preparation for 0–1 somite embryos

Embryos were dissociated and the resulting cell suspensions were processed separately for library preparation. Briefly, embryos were washed with Ca^+2^/Mg^+2^ free Dulbecco’s phosphate buffered saline (DPBS; Gibco, Life Technologies Corporation, Grand Island, NY, USA) to remove any FBS. Embryos were incubated in TrypLE™ Express (Gibco) at 37 °C for 6–7 min with intermittent pipetting using wide bore tips. Once embryos were completely dissociated, the enzyme was quenched using 10% FBS/DPBS (Ca^+2^/Mg^2+^ free) before the cells were resuspended in Ca^2+^/Mg^2+^ free DPBS containing 5% (v/v) endotoxin free FBS. Cell viability (i.e., lack of dead cells), number and size were confirmed with a hemocytometer. The single cell suspensions from individual embryos were loaded on a multiple-channel micro-fluidics chip of the Chromium Single Cell Instrument for GEM generation (10 × Genomics Inc., Pleasanton, CA, USA) with a targeted cell recovery of 5000–10000.

### scRNA-seq library preparation, sequencing, and alignment

The single cell suspension was processed using the 10 × Genomics Chromium Single Cell System (10 × Genomics, Inc.). Gene expression libraries were constructed using Chromium™ Single Cell 3’ Library and Gel Bead kit V3 (PN-120267), the Chromium™ Single Cell A Chip kit (PN-1000009) and Chromium™ i7 Multiplex Kit (PN-120262). Sequencing was performed by the Center for Medical Genomics (CMG) at Indiana University with the NovaSeq 6000 platform (Illumina, Inc., San Diego, CA, USA). Raw sequence data was processed with CellRanger 3.1.0 (10 × Genomics, Inc) and Bcl2fastq (https://support.illumina.com) transformed into sample-specific FASTQ files which were then aligned to the mouse reference genome mm10 (EGFP sequence was included) with the RNA-seq aligner STAR. The aligned reads were traced back to individual cells and the gene expression level of individual genes were quantified based on the number of UMIs (unique molecular indices) detected in each cell. The filtered feature-cell barcode matrices generated by CellRanger were used for further analysis.

### Quality control and clustering

Quality control analysis and clustering were performed with the R package Seurat version 4.0.4^43,44^. Briefly, the QC metrics of library size, number of features/genes, and mitochondrial reads were calculated. Cells with unique features/gene counts over 8500 (for the first and third embryo) or 7500 for the second embryo were deemed low quality and discarded. Cells with unique features/gene counts under 1500 and/or with a mitochondria gene percentage of over 10% were also discarded. After cells underwent an initial round of clustering, three clusters were removed due to cells with abnormally low mitochondrial percentage and feature counts, as well as overlap of markers of different cell subtypes (i.e., doublets or empty droplets). Then, the remaining cells were re-clustered, and the resulting clusters were annotated via expression of known marker genes. A heatmap and dot plot of the marker genes within these clusters were created with Seurat. The Seurat FindAllMarkers function identified gene expression markers for the resulting clusters. Loupe Browser 5.0.1 (10 × Genomics, Inc) was used for visualization and interactive examination of gene expression.

### RNAscope

Embryos were fixed in 4% (v/v) paraformaldehyde (PFA) diluted in PBS overnight at 4 °C. Embryos were then dehydrated through a methanol series and stored at − 20 °C for up to 3 weeks and rehydrated through a methanol series before performing the chromogenic *in-situ* hybridization assay using the RNAscope^®^ 2.5 High Definition—Brown kit (Advanced Cell Diagnostics, Newark, CA, USA) with some modifications to the manufacturer’s instructions. Briefly, embryos were not allowed to air-dry at any point. Embryos were first washed twice in PBST [PBS containing 0.1% (v/v) Tween-20], then bleached in 6% (v/v) hydrogen peroxide/PBS for 1 h and washed twice in PBST. After permeabilization with 10 µg/mL proteinase K for 45 s, embryos were incubated with 100 mM glycine followed by two washes in PBST. Embryos were then postfixed in 4% (v/v) PFA and 0.2% (v/v) glutaraldehyde diluted in PBS for 30 min at room temperature and washed twice in PBST. Embryos were washed once in PBS before an overnight incubation at 40 °C with 200 µL of one of the following RNAscope probes: *Mm-Sparcl1* (424641), *Mm-Syne1* (316511) and *Mm-Ttll3* (586791). Unless otherwise stated, embryos were washed twice with the Wash buffer (provided in the kit), 2 min each, at room temperature prior to incubation steps, and incubations were performed at 40 °C. Incubations for signal amplification and detection were carried out as follows: 400 µL of AMP1 for 30 min, 400 µL of AMP2 for 15 min, 400 µL of AMP3 for 30 min, 400 µL of AMP4 for 15 min, 400 µL of AMP5 at room temperature for 30 min, 400 µL of AMP6 at room temperature for 15 min, and a final incubation with 300 µL DAB-A/DAB-B at room temperature for 10 min. Embryos were then washed once with Wash buffer and once with PBS before imaging using a Nikon DS-Ri2 16MP digital camera attached to a Nikon SMZ1500 Zoom stereomicroscope (Nikon Inc., NY, USA).

### FACS sample preparation

Six *FOXJ1*-*EGFP* embryos ranging from 0 to 4 somites were collected and pooled to give 1086 EGFP^+^ LRO cells yielding 2.5 ng of RNA. A separate pooled sample, corresponding to the same LR patterning stages, was used for the collection of 12,000 surrounding EGFP^-^, non-LRO cells, yielding 206.7 ng of RNA. LRO cells were isolated from *FOXJ1*-EGFP embryos post micro-dissection and single cells in suspension were subjected to FACS as previously described^45^. EGFP^+^ and EGFP^-^ cells were sorted separately into RNA lysis buffer (Qiagen, Germantown, MD, USA). RNA purification was performed according to the RNeasy Micro kit protocol (Qiagen). Agilent RNA 6000 Pico Chip (Agilent Technologies, Santa Clara, CA, USA) was used to determine the quantity and quality of total RNA, yielding RNA integrity numbers greater than 9.0 (Supplementary Fig. S4a).

### Library preparation and analysis for bulk RNA-seq for mouse LRO and non-LRO cells

For the transgenic *FOXJ1-EGFP* mouse, the Ovation RNA-Seq System v2 (NuGEN, Tecan Group Ltd., Männedorf, Switzerland) was used to create double stranded cDNA from total RNA. The concentration of double stranded cDNA was calculated using the Qubit dsDNA BR Assay kit (Life Technologies). A DNA 1000 LabChip (Agilent Technologies) was used to analyze the size distribution of the cDNA. The cDNA library was prepared with the Nextera DNA Sample Preparation kit (Illumina) and the resulting library sequenced with an average of 40 million, 50 base-pair, single-end reads on an Illumina HiSeq 2500 platform according to Illumina protocols.

RNA-seq analysis follows the TopHat/Partek Genomics pipeline. All sequenced reads were mapped to the reference mouse genome using TopHat, which this aligns reads spanning known or novel splice junctions to create BAM files. The differential gene and transcript expression analysis was performed using RNA-seq workflow in Partek^®^ Genomics Suite^®^ software, version 6.6^©^ (2017 Partek Inc., St. Louis, MO, USA). Briefly, the BAM files were imported into Partek software for mRNA quantification. Differences in expression were determined by log-likelihood test that generated Chi-square and *p*-values at gene-level. Multiple test correction to reduce false discovery rate was applied using Partek GS RNA-seq workflow.

### Generation of LRO gene list and gene enrichment analysis

The LRO gene list was defined based on the following cut-offs: avg_log2FC > 0.4412, pct.1 > 0.374, pct.2 < 0.325 and pct.1–pct.2 > 0.347, where Pct.1 = percentage of cells in the LRO cluster that express the gene and Pct.2 = percentage of cells in the non-LRO clusters that express the gene. The cut-off, pct.1–pct.2 > 0.6 was used to identify the enriched genes when subclustering the LRO cluster. Enrichr^46^ was used for gene enrichment analysis.

## Supplementary Information

Supplementary Information.

Supplementary Tables.

## Data Availability

The datasets generated and/or analyzed during the current study have been deposited in the Gene Expression Omnibus (GEO) and are accessible through GEO Series accession number GSE212460.
