# Supplementary material for: Single cell RNA analysis of the left–right organizer transcriptome reveals potential novel heterotaxy genes
Source: Sci Rep. 2023 Jul 1;13:10688. doi: 10.1038/s41598-023-36862-2 (PMC10314903; doi:10.1038/s41598-023-36862-2)
Supplement: Supplementary file 1 — Supplementary Information. [file 41598_2023_36862_MOESM1_ESM.pdf]

Single cell RNA analysis of the left-right organizer transcriptome reveals potential novel heterotaxy genes

Helen M. Bellchambers[1,†], Amruta R. Phatak[1,†], Mardi J. Nenni[2], Maria B. Padua[1], Hongyu Gao[3], Yunlong Liu[3], Stephanie M. Ware [1,3,*]

[1]Herman B Wells Center for Pediatric Research, Department of Pediatrics, Indiana University School of Medicine, Indianapolis, IN, 46202, USA

[2]Division of Developmental Biology, Cincinnati Children's Hospital Medical Center, Cincinnati, OH, 45229, USA

[3]Department of Medical and Molecular Genetics, Indiana University School of Medicine, Indianapolis, IN, 46202, USA

[†] These authors contributed equally to this work

* Corresponding author

Supplementary Methods – Annotation of cell types

To determine the cluster identities, the cells were first roughly divided into three major cell lineages: ectoderm, mesoderm, and endoderm. Clusters were identified as endoderm cells by expression of the general endoderm markers *Spink1, Sox17* and *Foxa2*. Within these clusters, two mesendoderm clusters were identified by overlapping expression of the endoderm marker *Foxa2* and the mesoderm marker *T*. The annotation of these mesendoderm clusters into left-right organizer and axial mesoderm is described in detail in the main text. Extraembryonic endoderm was identified by expression of several markers including *Ttr*, *Apoa2*, *Apoa1*, *Apoe*, *Apoc1a* and *Afp*. The remaining endoderm cluster was identified as definitive endoderm by expression of the marker *Apela* and absence of the other endoderm markers.

Ectoderm clusters were identified by expression of the general ectoderm markers *Sox2* and *Dlx5*. Some cells were shown to have expression of both general ectoderm markers and mesoderm markers, based on expression of both *Sox2* and *T,* which has previously been described for the bi-potent neuromesodermal progenitors (NMP). These appear to be cells that remain capable of forming either ectoderm or mesoderm (i.e., epiblast cells). One of these clusters also showed expression of the caudal marker *Wnt3a* and was thus defined as NMP/caudal epiblast. The other cluster showed expression of several anterior markers including *Otx2*, *Six3* and *Hesx1* and so was defined as NMP/anterior epiblast. The remaining ectoderm clusters were identified as neuroectoderm by expression of *Sox1* and *Pou3f1* or as surface ectoderm by expression of *Wnt6*.

Of the remaining clusters, the mesenchyme cells were identified by expression of *Bmp4* and *Ahnak*, the allantois clusters were identified by expression of *Hoxa10*, *Hoxa11*, and *Tbx4*, the erythroid cells were identified by expression of *Hbb-bh1*, *Cited4* and *Gypa* whereas the endothelium cells were identified by expression of *Kdr* and *Esam*. The remaining three clusters showed expression of one or more mesoderm markers. Of these, one was identified as presomitic mesoderm due to expression of many presomitic markers including *Tbx6*, *Dll1*, *Dll3*, *Msgn1*, *Hes5*, *Hes7*, *Rspo3* and *Lfng*.

One of the remaining clusters appears to be a mix of two different cell types. All cells within the cluster showed expression of *Isl1* and *Nkx2-5*. A portion of the cells within this cluster showed expression of multiple cardiac mesoderm markers including *Tnnt2*, *Myl4*, and *Myl7*. The other cells in the cluster showed expression of *Tbx1*, which is known to mark the cardiopharyngeal mesoderm when its expression overlaps with *Isl1* or *Nkx2-5*. We thus termed this cluster the cardiac and pharyngeal mesoderm cluster.

The remaining cluster showed expression of *Tbx1*, which marks the head mesoderm as well as *Tcf15, Meox1, Aldh1a2a* and *Foxc1* which are markers of the presomitic/somitic mesoderm. These cells were labelled paraxial mesosderm, a term which includes both the somitic and head mesoderm.

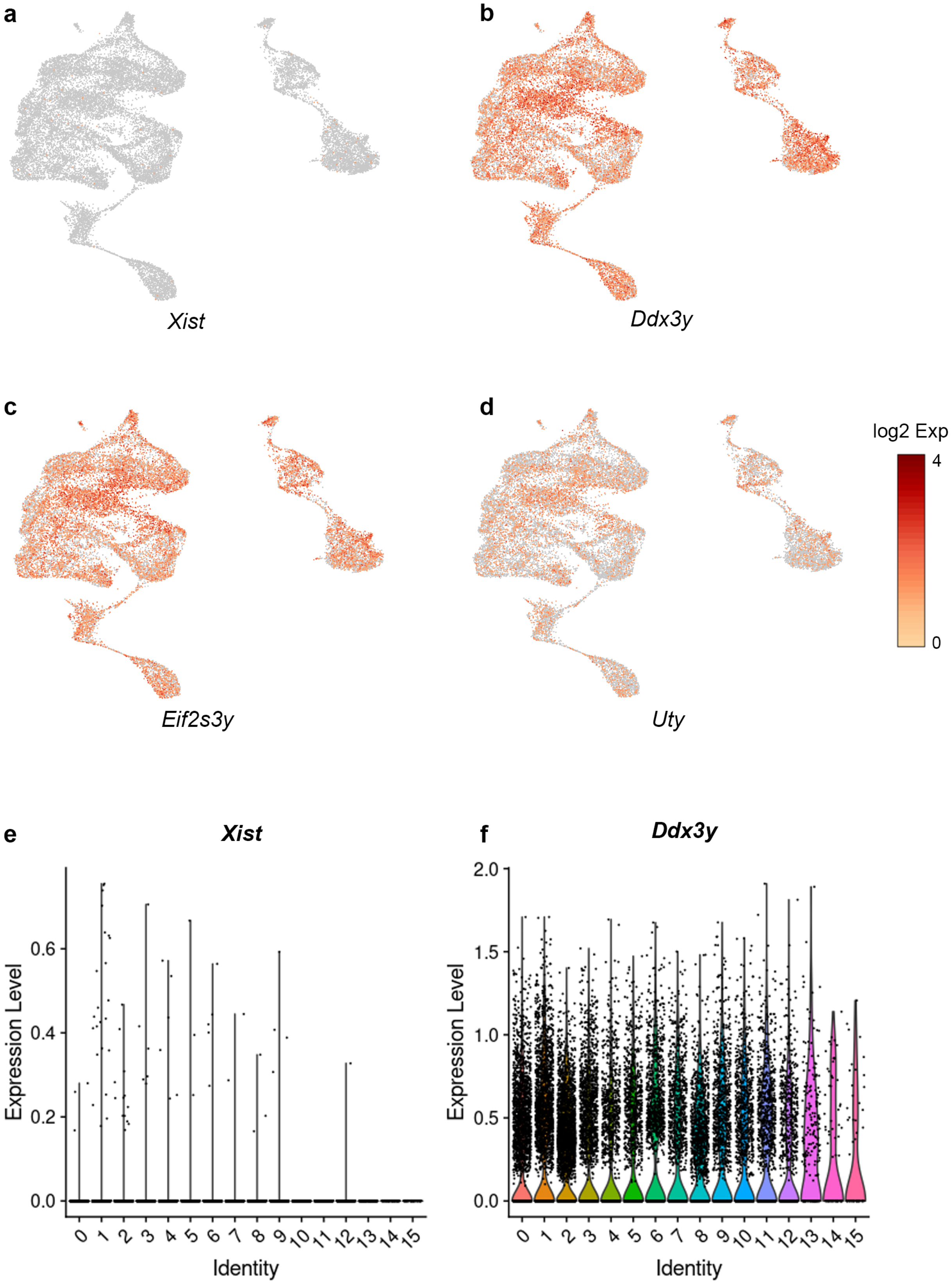

Supplementary Figure S1

Supplementary Figure S1: Expression of female and male specific genes. (a) Expression of the female cell specific transcript *Xist* showing the lack of female cells indicating no maternal contamination. b-d) Expression of Y chromosome genes including (b) *Ddx3y*, (c) *Eif2s3y* and (d) *Uty*. e-f). Violin plots of (e) the female cell specific transcript *Xist* expression and (f) the Y chromosome gene *Ddx3y* expression in the clusters. Each dot represents the log normalized expression value (y-axis) of a single cell of a particular cluster (x-axis). Cluster identities are listed in Supplementary Table 3.

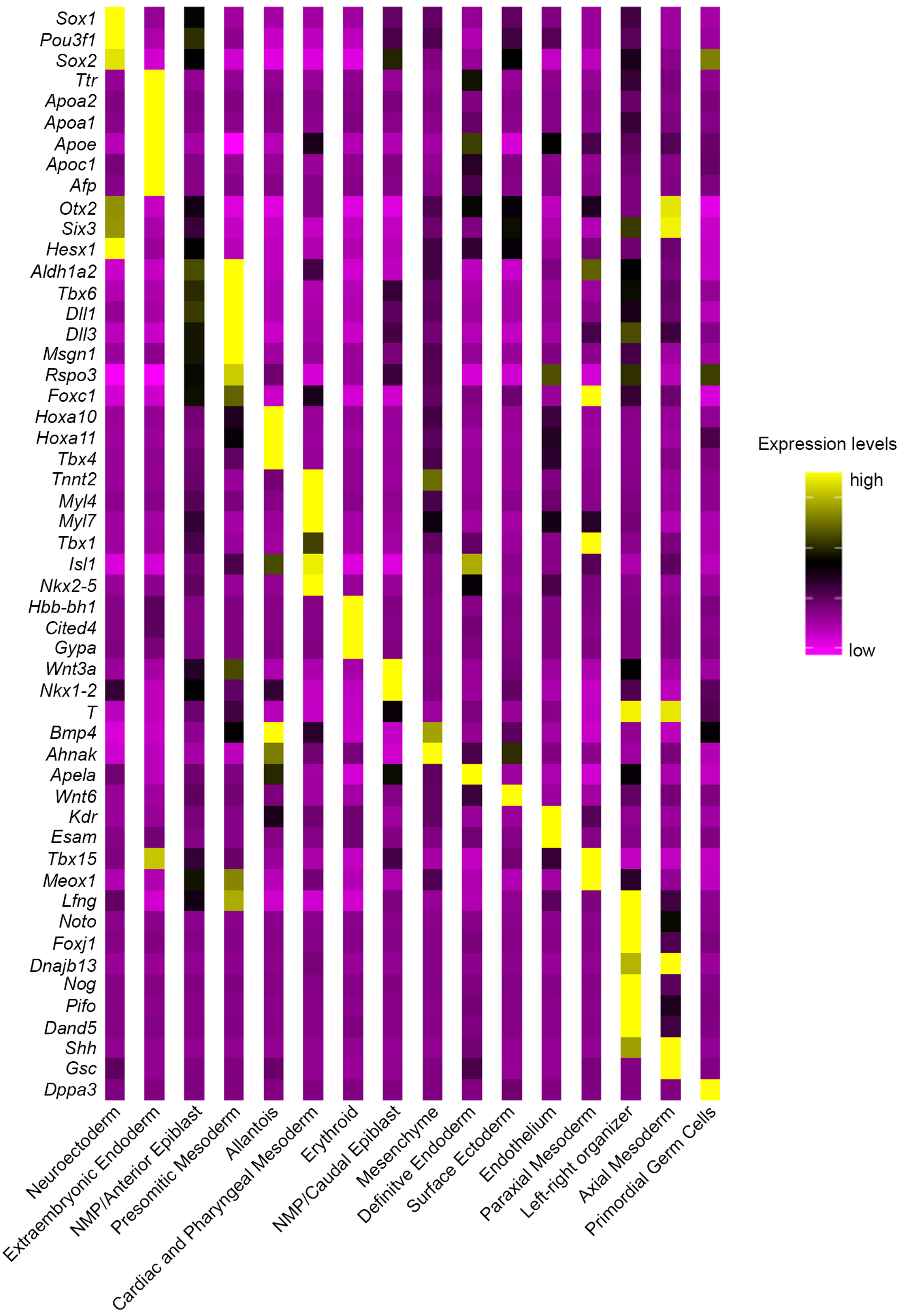

Supplementary Figure S2

Supplementary Figure 2: Heatmap of known marker genes for 16 distinct cell clusters. Marker genes are listed on the y-axis and cluster identities are on the x-axis. Color indicates the average expression level of the gene, with yellow indicating the clusters' highest expression of the gene. NMP, Neuromesodermal progenitors

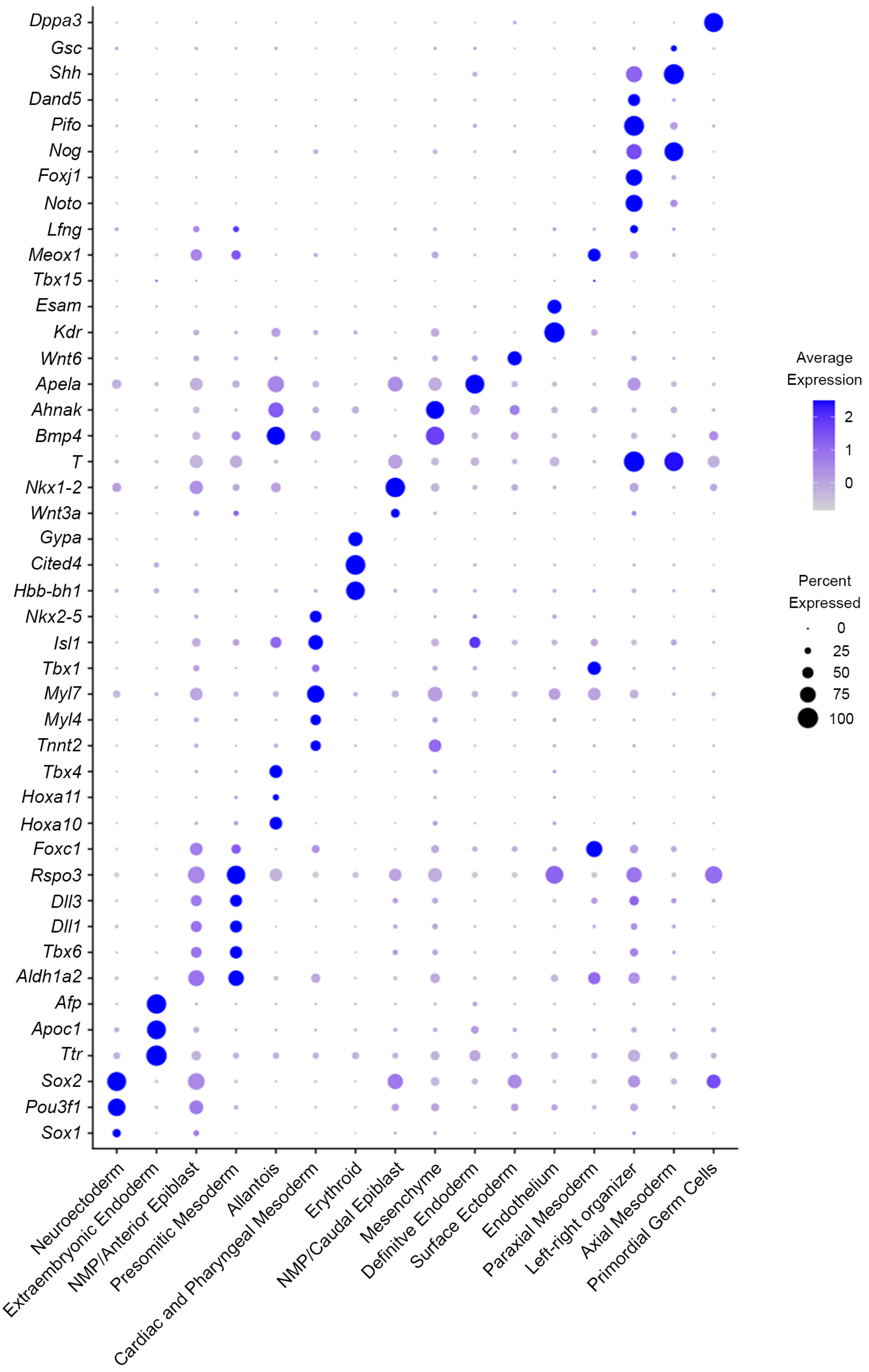

Supplementary Figure S3

Supplementary Figure 3: Dot plot of known marker genes for 16 distinct cell clusters. Marker genes are listed on the y-axis and cluster identities are on the x-axis. Dot size represents the percentage of cells in each cluster expressing the gene (the larger the dot the higher proportion of cells expressing the gene). Dot color indicates the average expression level of gene within the cluster. NMP, Neuromesodermal progenitors

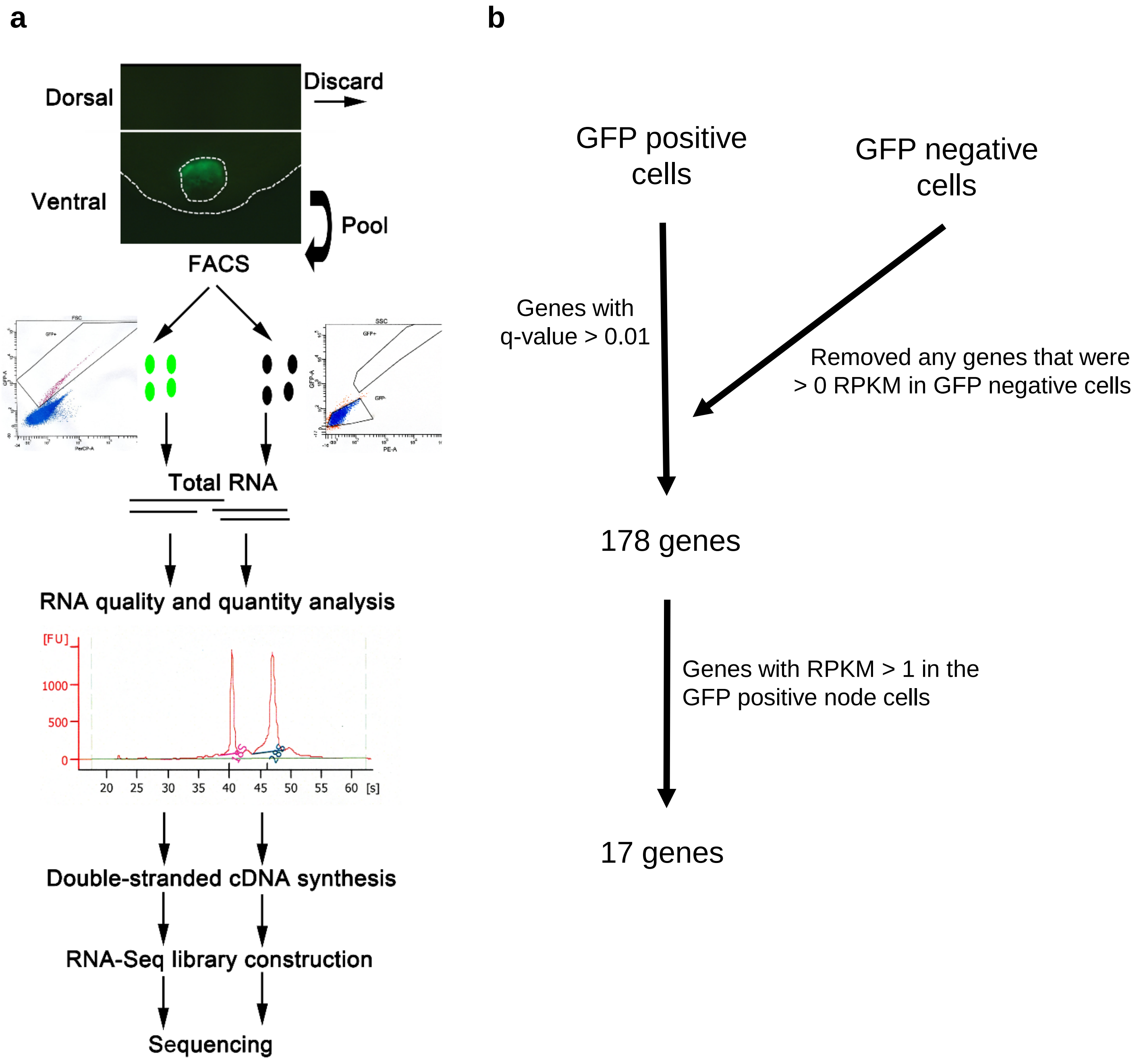

Supplementary Figure S4

Supplementary Figure 4: Overview of the methods used to sequence total RNA from pooled samples of left-right organizer (LRO) and surrounding non-LRO cells. (a) Micro-dissection of *FOXJ1-EGFP* embryos enriched for LRO cells and surrounding non-LRO cells. FACS isolated GFP-positive LRO cells from surrounding GFP-negative non-LRO cells. Total RNA was extracted and subjected to quality control analyses. RNA meeting quality control standards was converted to double-stranded cDNA, amplified and an RNA-seq library was constructed for sequencing. (b) Schematic overview of filtering methods utilized to define LRO specific genes and LRO-enriched genes. Using Partek Genomics Suite Software, the genes from GFP-positive cells were filtered to remove genes with a q-value greater than 0.1 as well as genes expressed in the GFP negative cells. The remaining genes where further filtered to limit the list to genes with reads per kilobase per million (RPKM) expression greater than 1, which left 17 LRO-specific genes.

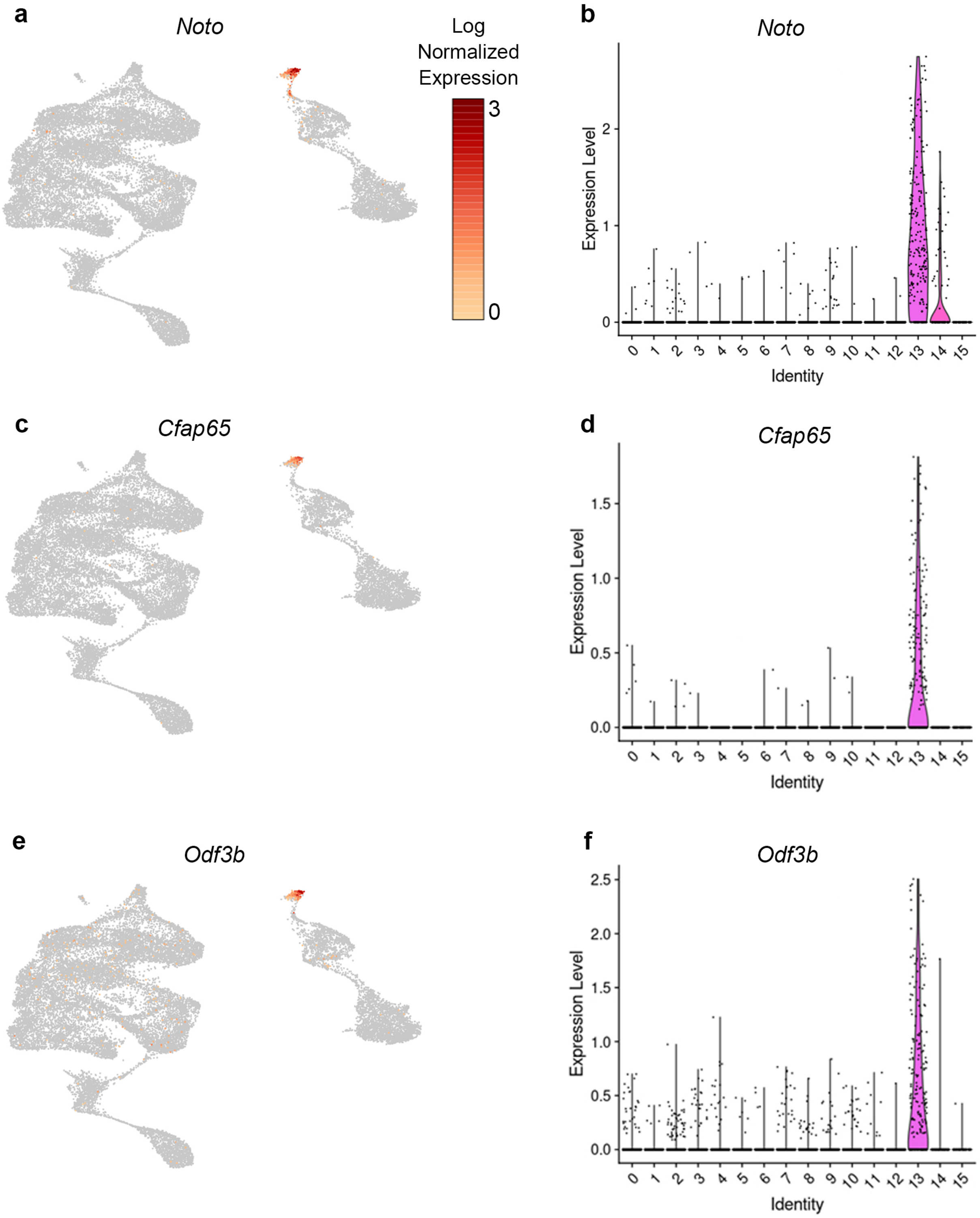

Supplementary Figure S5

Supplementary Figure 5: Expression of left-right organizer (LRO) genes detected by fluorescent activated cell sorting (FACS) and bulk RNA-seq. (a, c, e) Feature plots and (b, d, f) violin plots of (a-b) *Noto*, (c-d) *Cfap65* and (e-f) *Odf3b*, which are three of the genes identified as LRO specific by bulk RNA-seq of FACS sorted cells. For the violin plots, each dot represents the log normalized expression value (y-axis) of a single cell of a particular cluster (x-axis). Cluster identities are listed in Supplementary Table 3.
